# Intrinsic Capacity And Longevity From Age 70-100

**DOI:** 10.1093/geroni/igaf122.164

**Published:** 2025-12-31

**Authors:** Jeremy Jacobs, Irit Stessman-Lande, Aliza Rozenberg, Jochanan Stessman

**Affiliations:** Hadassah Hebrew University Hospital, Jerusalem, Yerushalayim, Israel; Hadassah University Medical Centers; The Department of Geriatric Rehabilitation, Jerusalem, Yerushalayim, Israel; Hebrew University of Jerusalem, Hadassah Medical Center, Jerusalem, Yerushalayim, Israel; Hebrew University of Jerusalem, Hadassah Medical Center Faculty of Medicine, Jerusalem, Yerushalayim, Israel

## Abstract

Intrinsic capacity (IC) has been proposed as a useful marker of healthy aging. This study examines the prevalence of IC between ages 70-100, and its association with survival. The Jerusalem Longitudinal Study (1990-2021) prospectively followed a community-dwelling cohort born 1920-21, at ages 70, 78, 85, 90, 95 and 100 (n = 604, 1024, 1222, 729, 508, 205 respectively). Comprehensive assessment was performed, and mortality data collected. Intrinsic capacity (0-100/minimum-maximum) was a composite of Cognition (Mini-Mental State Exam), Psychological (depression using the Basic Symptom Inventory), Vitality (BMI), Locomotion (chair transfer) and Sensory (Snellen and whisper test) domains. Kaplan-Meier survival curves, and Cox Proportional Hazard models were performed. The mean IC at ages 70, 78, 85, 90, 95, and 100 years consistently declined: 75.8, 74.0, 66.7, 64.5, 63.5, 51.8, and was higher in males vs. females: 75.4/72.5, 77.1/74.1, 69.0/64/8, 68.7/61.1, 64.3/62.9, 53.1/51.3. A consistent pattern of age-dependent decline was observed within each separate domain. Using various cut-off points for IC, increased IC was consistently associated with improved survival throughout follow-up, except for age 78-85. Treating IC as a continuous variable, after adjusting for gender, education and Charlson comorbidity index, the mortality Hazard Ratios at age 70, 78, 85, 90, and 95 were 0.98 (95%CI 0.95-1.0, p = 0.039), 1.0 (95%CI 0.98-1.02, p = 0.91), 0.98 (95%CI 0.97-0.99, p < 0.01), 0.98 (95%CI 0.96-0.99, p < 0.01), 0.99 (95%CI 0.98-1.0, p = 0.19). Using a simple operational definition, our findings describe the consistent trajectory of age-related decline in Intrinsic Capacity between age 70-100, and support its usefulness as a marker for subsequent longevity.

